# A Novel A-ECMS Energy Management Strategy Based on Dragonfly Algorithm for Plug-in FCEVs

**DOI:** 10.3390/s23031192

**Published:** 2023-01-20

**Authors:** Shibo Li, Liang Chu, Jincheng Hu, Shilin Pu, Jihao Li, Zhuoran Hou, Wen Sun

**Affiliations:** 1 College of Automotive Engineering, Jilin University, Changchun 130022, China; 2 Department of Aeronautical and Automotive Engineering, Loughborough University, Loughborough LE11 3TU, UK; 3 College of Automotive Engineering, Changzhou Institute of Technology, Changzhou 213032, China

**Keywords:** adaptive equivalent consumption minimization strategy (A-ECMS), dragonfly algorithm (DA), 4-wheel-drive plug-in fuel cell electric vehicles (4WD PFCEVs), energy management strategy (EMS)

## Abstract

The mechanical coupling of multiple powertrain components makes the energy management of 4-wheel-drive (4WD) plug-in fuel cell electric vehicles (PFCEVs) relatively complex. Optimizing energy management strategies (EMSs) for this complex system is essential, aiming at improving the vehicle economy and the adaptability of operating conditions. Accordingly, a novel adaptive equivalent consumption minimization strategy (A-ECMS) based on the dragonfly algorithm (DA) is proposed to achieve coordinated control of the powertrain components, front and rear motors, as well as the fuel cell system and the battery. To begin with, the equivalent consumption minimization strategy (ECMS) with extraordinary instantaneous optimization ability is used to distribute the vehicle demand power into the front and rear motor power, considering the different motor characteristics. Subsequently, under the proposed novel hierarchical energy management framework, the well-designed A-ECMS based on DA empowers PFCEVs with significant energy-saving advantages and adaptability to operating conditions, which are achieved by precise power distribution considering the operating characteristics of the fuel cell system and battery. These provide state-of-the-art energy-saving abilities for the multi-degree-of-freedom systems of PFCEVs. Lastly, a series of detailed evaluations are performed through simulations to validate the improved performance of A-ECMS. The corresponding results highlight the optimal control performance in the energy-saving performance of A-ECMS.

## 1. Introduction

In the context of global issues such as energy scarcity and global warming, hydrogen energy is being favored by the automotive industry as a sustainable and clean energy source [1,2]. In the global automotive companies’ vigorous electrification transition, PFCEVs have become an alternative to conventional internal combustion engine vehicles thanks to their considerable environmental friendliness, high efficiency, and competitive range [3,4]. As a nonlinear complex system with multiple power and energy degrees of freedom, 4WD PFCEVs urgently need a refinement-designed EMS to improve the economy through the rational distribution of power under different operating conditions [5,6]. However, proposing an EMS adaptable to operating conditions for such nonlinear complex systems is still a pressing problem.

Existing EMSs for PFCEVs can be classified into three categories: rule-based EMSs (RB-EMSs), optimization-based EMSs (OB-EMSs), and learning-based EMSs (LB-EMSs) [7,8,9]. Rule-based EMS generally establishes rules through human inspiration and expert experience, such as state flow diagrams [10,11] or fuzzy rules [12]. Therefore, RB-EMSs can complete rule formulation and real-time applications without a priori knowledge of driving cycles. However, although RB-EMSs have superior robustness, the primarily deterministic rules cannot adaptively adjust to the driving cycles. Furthermore, OB-EMSs can be further classified as global OB-EMSs and instantaneous OB-EMSs [13,14]. As a typical global OB-EMS, dynamic programming (DP) simplifies the optimization problem into a multi-step decision process by recursion based on the Bellman equation [15,16]. Therefore, a priori knowledge of the complete driving cycles and computational resources with superior performance are necessary for DP to achieve optimal control. Thus, DP can only be used as a baseline. As another global OB-EMS, Pontryagin’s minimum principle (PMP) determines the optimal control trajectory by iterative search [12,17]. However, PMP is an indirect method to obtain global optimization results, which cannot guarantee the optimal global solution, and PMP is also more challenging to apply directly to real-time control. In contrast, instantaneous OB-EMSs require only partial a priori knowledge of short future driving cycles to solve the local optimal control trajectory at a particular moment [18,19], receiving widespread attention from researchers. Model predictive control (MPC) [20,21,22] can solve the local optimal control trajectory in a particular horizon, taking into account control performance and real-time application ability, and the adaptability towards operating conditions can be achieved through the accurate generation of reference trajectories. However, the control performance of MPC is deeply affected by the modeling accuracy of the state observation model and the reference trajectory prediction model, so it is still a challenge to establish a high-precision model for the highly nonlinear system of 4WD PFCEVs’ powertrain. The equivalent energy consumption minimization strategy (ECMS) [23,24] can solve the optimal power distribution at a particular instantaneous moment, which has a superior instantaneous optimal performance and real-time application ability. However, the equivalent factors in ECMS that determine the energy conversion of different energy sources are not well determined, which will affect the adaptability of ECMS to operating conditions. LB-EMSs, such as reinforcement learning [25,26] and rule-based learning [1], are strongly adaptive. Although they do not require absolute model knowledge, creating an accurate database that directly impacts control performance is difficult and time-consuming. Additionally, it is prone to local convergence under different action constraints. Therefore, an ECMS with optimized equivalent factors can provide more accurate instantaneous optimization and real-time applications, providing better energy-saving solutions for PFCEVs.

To enhance the comprehensive performance of an ECMS, a series of adaptive ECMS (A-ECMS) approaches have been proposed to improve control performance and operating condition adaptability by optimizing the equivalent factors. In ongoing research, A-ECMS can predict future information on vehicle velocity and demand power based on current and historical data, including reinforcement learning [27], BP neural networks [28], artificial neural networks (ANN) [29], learning vector quantization (LVQ) [30], chaining neural networks (CNN) [31], and iterative learning [32,33]. In these cases, the equivalent factor is optimized in advance. Nevertheless, the future velocity information is variable in time and space under complex and changing operating conditions. Establishing a high-precision model to predict future velocity information accurately is more challenging. Even though the above methods have an excellent velocity prediction under particular operating conditions, the computational demand is increased significantly, which leads to inferior real-time application abilities. Therefore, the implementation process of A-ECMS must reduce the unreasonable adjustment of equivalent factors due to inaccurate future a priori information, which is considered a critical challenge to be addressed.

This paper proposes a novel A-ECMS EMS based on the DA (dragonfly algorithm). In the meticulously designed A-ECMS, the front and rear motor co-distribution is accomplished based on ECMS, aiming at achieving optimal drive control under different vehicle demand power and motor characteristics. In addition, a novel hierarchical control framework for SOC-based EMS is proposed to realize the coordinated power output of the fuel cell system and the battery based on A-ECMS. To further exploit the vehicle’s energy-saving potential and optimize the adaptation to operating conditions, the dragonfly algorithm is employed to optimize the equivalent factor vector of A-ECMS. The simulation comparison validates the effectiveness of the proposed strategy. Accordingly, the contributions provided are:

(1) An A-ECMS for 4WD PFCEVs that can fully exploit the energy-saving potential of powertrain components and improve vehicle economy.
(2) A novel hierarchical energy management framework based on battery SOC is proposed to improve the ability of precise control and the cooperative response of the powertrain.
(3) Considering the different characteristics of front and rear motors and real-time power requests, ECMS-based power distribution of front and rear motors (M-ECMS) is employed in 4WD PFCEVs to optimize motor operating states and achieve optimal drive control.
(4) Based on the dragonfly algorithm, the equivalence factors of A-ECMS are optimized to exploit the vehicle’s energy-saving potential and optimize the adaptation to operating conditions through coordinated control of the fuel cell system and the battery.

The rest of the paper is organized as follows: Section 2 introduces the modeling of the PFCEV. Section 3 presents the energy management strategy of A-ECMS based on the dragonfly algorithm. Simulations and discussions are described in Section 4. Finally, conclusions are drawn in Section 5.

## 2. Models

### 2.1. Powertrain Configuration

**Figure 1:**
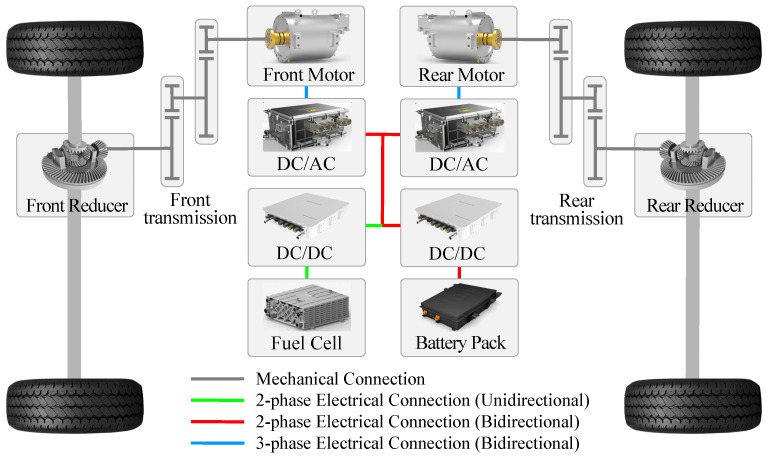
Powertrain configuration of the studied PFCEV.

Figure 1 illustrates the powertrain configuration of the studied PFCEV, where a fuel cell stack and a battery pack are connected to the DC bus via a unidirectional DC/DC and a bidirectional DC/DC, respectively, providing energy to the front and rear motors via two DC/AC inverters. Furthermore, the front motor is connected to the front wheels through a front transmission and a front reducer, while the rear motor is connected similarly to the front one. Specifically, the basic parameters of the PFCEV are listed in Table 1. Note that the studied PFCEVs include pure electric vehicle (EV) and hybrid electric vehicle (HEV) modes. In HEV mode, the fuel cell system and the battery jointly respond to the vehicle demand power.

### 2.2. Rule-Based Hierarchical Energy Management Framework

**Figure 2:**
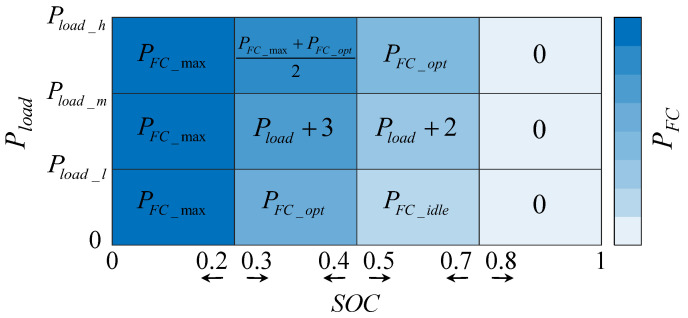
Rule-based energy management framework.

As a baseline, the original vehicle uses a rule-based hierarchical EMS with the control framework shown in Figure 2. In particular, the framework is divided into four levels based on the battery SOC: high SOC, relatively high SOC, relatively low SOC, and low SOC. In order to finely distribute the output power of the fuel cell system and the battery, the two levels of relatively high SOC and relatively low SOC are further divided into high, medium, and low levels according to the vehicle demand power, respectively. The twelve zones divided by four levels of SOC and three levels of vehicle demand power represent one operating state of the fuel cell system, i.e., the fuel cell system output power, where SOC is the battery SOC, $P_{load}$ is the vehicle demand power, and $P_{FC}$ is the fuel cell system demand power. $P_{load\_h}$ is the high constant of vehicle demand power, $P_{load\_m}$ is the medium constant of vehicle demand power, and $P_{load\_l}$ is the low constant of vehicle demand power. $P_{FC\_\max}$ is the maximum fuel cell system power, $P_{FC\_opt}$ is the power corresponding to the highest efficiency point of the fuel cell system, and $P_{FC\_idle}$ is the fuel cell system idle power.

### 2.3. Powertrain Modeling

The vehicle dynamics equation for a PFCEV when driving on a flat road with sufficient traction can be described as follows:

(1) $$F_{loadM}=mgf+\frac{1}{2}C_{D}AV^{2}+\delta m\frac{dV}{dt}$$

Further, the vehicle driving power equilibrium equations can be written as follows:

(2) $$P_{loadM}=\left(mgf+\frac{1}{2}C_{D}AV^{2}+\delta m\frac{dV}{dt}\right)V$$

where $F_{loadM}$ is the vehicle driving force, $P_{loadM}$ is the vehicle driving power, *m* is the vehicle mass, *g* is the gravitational acceleration, *f* is the rolling resistance coefficient, $C_{D}$ is the air drag coefficient, *A* is the vehicle frontal area, *V* is the vehicle velocity, and δ is the weighting factor of the rotating mass.

Therefore, the vehicle demand power $P_{load}$ can be defined as follows:

(3) $$\left\{\begin{array}{c} P_{load}=\frac{P_{FM}}{\eta_{FMotor}}+\frac{P_{RM}}{\eta_{RMotor}}\\ P_{FM}=u_{Motor}\cdot P_{loadM}\\ P_{RM}=\left(1-u_{Motor}\right)\cdot P_{loadM} \end{array}\right.$$

where $P_{FM}$ and $P_{RM}$ are the mechanical power of the front and rear motors, respectively; $u_{Motor}$ is the power distribution coefficient between the front motor and rear motors; and $\eta_{FMotor}$ and $\eta_{RMotor}$ are the efficiency of the front and rear motors, respectively. The power of the dual energy source should satisfy the following equation:

(4) $$\left\{\begin{array}{c} P_{FC}=u_{ES}\cdot P_{load}\\ P_{Batt}=\left(1-u_{ES}\right)\cdot P_{load} \end{array}\right.$$

where $P_{FC}$ is the output power of the fuel cell system, $P_{Batt}$ is the battery power, and $u_{ES}$ is the power distribution coefficient between the fuel cell system and the battery.

#### 2.3.1. Fuel Cell Modeling

**Figure 3:**
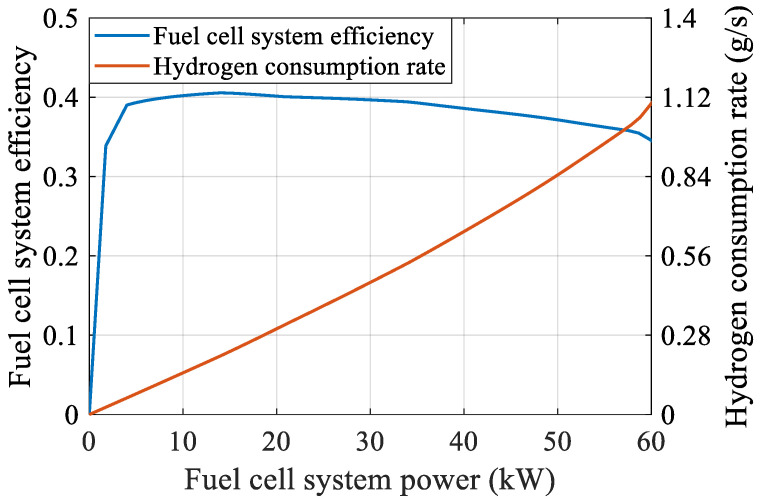
Fuel cell system efficiency and hydrogen consumption rate.

A proton exchange membrane fuel cell (PEMFC) is used in this study. Since the fuel cell is a nonlinear and complex system, the characteristics of this complex nonlinear system are easily affected by the operating environment. Therefore, a static model of the fuel cell system is obtained from experimental data under the assumption of constant ambient temperature and gas pressure. Figure 3 illustrates the details of the static model of the fuel cell system, including the relationship between the fuel cell system power and efficiency, as well as the relationship between the fuel cell system power and hydrogen consumption rate.

Theoretically, the hydrogen consumption of a fuel cell system is related to the efficiency of the fuel cell system as follows:

(5) $$m_{FCH_{2}}=\frac{1}{LHV_{H_{2}}}\int \frac{P_{FC}}{\eta_{FC}\left(P_{FC}\right)}$$

In this study, the hydrogen consumption of the fuel cell system is obtained by interpolating and integrating the characteristic hydrogen consumption rate curve shown in Figure 3, which is written as follows:

(6) $$m_{FCH_{2}}=\int P_{FC}\cdot C_{H_{2}}\left(P_{FC}\right)$$

where $m_{FCH_{2}}$ is the hydrogen consumption of the fuel cell system, $LHV_{H_{2}}$ is the hydrogen low calorific value, here taken as 120 MJ/kg, and $\eta_{FC}$ and $C_{H_{2}}$ are the fuel cell system efficiency and hydrogen consumption rate, which are functions of the fuel cell system power.

#### 2.3.2. Battery Modeling

Batteries have the advantage of high energy density but low power density. Moreover, due to internal resistance, the environment easily affects the charging and discharging performance, especially the temperature. Similar to fuel cells, batteries are also a highly nonlinear system. Taking into account factors such as modeling accuracy and computational resource requirements, this study establishes a first-order resistance-capacitance (RC) model from experimental data, in which both the open circuit voltage and the charging/discharging internal resistance of the battery are functions of SOC and temperature. Therefore, the battery terminal voltage can be written as a function of open circuit voltage and internal resistance as follows:

(7) $$U_{Batt}=U_{BattOCV}-R_{Batt}I_{Batt}$$

where $U_{Batt}$ is the battery terminal voltage, $U_{BattOCV}$ is the battery open circuit voltage, $R_{Batt}$ is the battery internal resistance, and $I_{Batt}$ is the battery current. Define $I_{Batt}>0$ for battery discharge.

Further, the battery current and the battery SOC can be written as a function of the battery power [1] as follows:

(8) $$I_{Batt}=\frac{U_{BattOCV}-\sqrt{U_{BattOCV}^{2}-4R_{Batt}P_{Batt}}}{2R_{Batt}}$$

(9) $$S\dot{O}C=-\frac{U_{BattOCV}-\sqrt{U_{BattOCV}^{2}-4R_{Batt}P_{Batt}}}{2R_{Batt}C_{Batt}}$$

where $P_{Batt}$ is the battery power and defines the battery discharging process as $P_{Batt}>0$. $C_{Batt}$ is the battery capacity.

Battery efficiency is defined as the ratio of battery power to total power, which is calculated as follows:

(10) $$\eta_{Batt}=\left\{\begin{array}{c} \frac{P_{Batt}}{P_{Batt}+P_{BattLoss}}=\frac{U_{Batt}I_{Batt}}{U_{Batt}I_{Batt}+I_{Batt}^{2}R_{Batt}},\;P_{Batt}>0\\ \frac{P_{Batt}+P_{BattLoss}}{P_{Batt}}=\frac{U_{Batt}I_{Batt}+I_{Batt}^{2}R_{Batt}}{U_{Batt}I_{Batt}},\;P_{Batt}<0 \end{array}\right.$$

where $\eta_{Batt}$ is the battery efficiency and $P_{BattLoss}$ is the battery power loss due to internal resistance. Note that the battery efficiency is set to 1 when $P_{Batt}=0$.

#### 2.3.3. Motor Modeling

**Figure 4:**
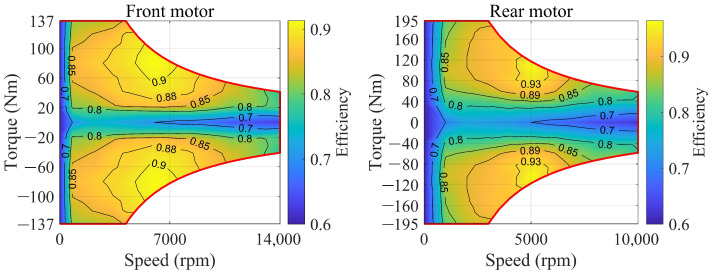
Front and rear motor efficiency.

The front and rear axles of the studied PFCEV are each equipped with a permanent magnet synchronous motor (PMSM), which has the advantages of high efficiency, low-temperature rise, and high overload capacity. The efficiency of the motor can be obtained by looking up the map of motor torque and speed when the thermal and dynamic performance of the motor are neglected, as shown in Figure 4. Therefore, the motor efficiency can be written as:

(11) $$\left\{\begin{array}{c} \eta_{FMotor}=f_{FMotor}\left(T_{FMotor},n_{FMotor}\right)\\ \eta_{RMotor}=f_{RMotor}\left(T_{RMotor},n_{RMotor}\right) \end{array}\right.$$

where $\eta_{FMotor}$ and $\eta_{RMotor}$ are the efficiency of the front and rear motors, respectively, $T_{FMotor}$ and $T_{RMotor}$ are the torque of the front and rear motors, respectively, and $n_{FMotor}$ and $n_{RMotor}$ are the speed of the front and rear motors, respectively. Furthermore, the mechanical power of the front and rear motors, as well as the electric power of the front and rear motors, can be written as follows:

(12) $$\left\{\begin{array}{c} P_{FM}=\frac{T_{FMotor}\cdot n_{FMotor}}{9550}\\ P_{RM}=\frac{T_{RMotor}\cdot n_{RMotor}}{9550} \end{array}\right.$$

(13) $$\left\{\begin{array}{c} P_{FE}=\left\{\begin{array}{c} \frac{P_{FM}}{\eta_{FMotor}},\mathrm{Driving}\\ P_{FM}\eta_{FMotor},\mathrm{Braking} \end{array}\right.\\ P_{RE}=\left\{\begin{array}{c} \frac{P_{RM}}{\eta_{RMotor}},\mathrm{Driving}\\ P_{RM}\eta_{RMotor},\mathrm{Braking} \end{array}\right. \end{array}\right.$$

where $P_{FM}$ and $P_{RM}$ represent the mechanical power of the front and rear motors, respectively, and $P_{FE}$ and $P_{RE}$ represent the electric power of the front and rear motors, respectively.

## 3. A-ECMS EMS Based on Dragonfly Algorithm

**Figure 5:**
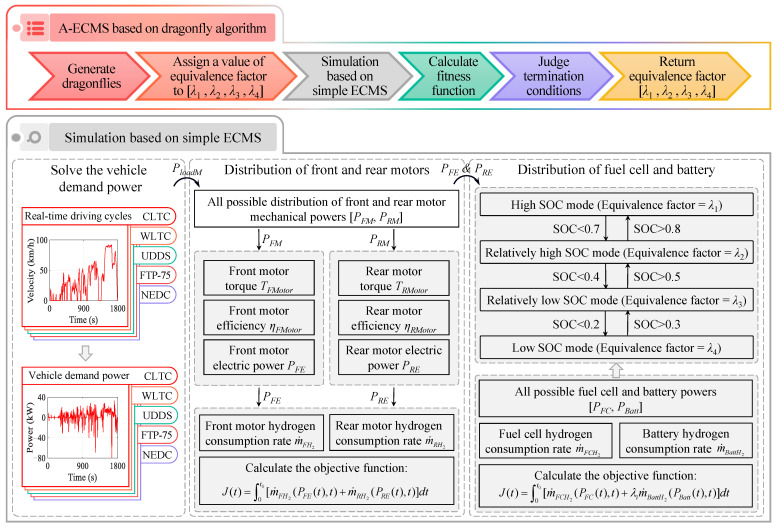
A-ECMS EMS based on dragonfly algorithm.

This section presents the A-ECMS EMS based on the dragonfly algorithm, as shown in Figure 5. Under the expected driving conditions of the vehicle, the front and rear motors of the 4WD PFCEV are coupled to the ground, i.e., the front and rear motor speeds are in fixed proportion to the vehicle’s velocity. Meanwhile, since the efficiency characteristics of the front and rear motors are distinctly different, their power distribution directly affects the operating point and the vehicle economy. In addition, since the fuel cell system and the battery also possess different characteristics, their operating conditions are equally crucial for vehicle economy. Accordingly, this study distributes the power between the front and rear motors based on a simple ECMS. Moreover, the power of the fuel cell and the battery is subsequently distributed based on A-ECMS, whose equivalent factors are iteratively optimized based on the dragonfly algorithm. Consequently, the economy of the 4WD PFCEV is improved while ensuring the above powertrain components operate at efficient points.

### 3.1. Simple ECMS

ECMS is developed from PMP for solving the optimal solution of continuous or discontinuous control systems. Specifically, ECMS can solve for the optimal control inputs that satisfy the minimum cost function after a given set of boundary conditions, aiming to minimize the Hamilton equation. A controlled system can be expressed as the following mathematical equation:

(14) $$\dot{x}\left(t\right)=f\left(x,u,t\right)$$

where *x* represents the state variables and *u* represents the control inputs. In addition, the cost function of the controlled system can be described as follows:

(15) $$J\left(t\right)=\int_{0}^{t_{0}}C(x(t),u(t),t)dt$$

where C(x(t),u(t),t) is the instantaneous cost. To solve for the minimal cost function of the controlled system, the corresponding Hamilton equation can be written as follows:

(16) $$H\left(x\left(t\right),u\left(t\right),\kappa \left(t\right),t\right)=C\left(x\left(t\right),u\left(t\right),t\right)+\kappa {\left(t\right)}^{T}f\left(x\left(t\right),u\left(t\right),t\right)$$

where κ(t) is the co-state variable. Therefore, under certain constraints, the optimal solution can be obtained by satisfying the minimum of the Hamilton function in a finite set, which can be written as follows:

(17) $$u^{*}\left(t\right)=\arg\min_{u}\;\left[H\left(x\left(t\right),u\left(t\right),\kappa \left(t\right),t\right)\right]$$

where $u^{*}\left(t\right)$ is the optimal solution obtained by ECMS.

In the energy management of the 4WD PFCEV, in order to achieve a more competitive total hydrogen consumption, the Hamilton equation can be set to the total hydrogen consumption of either the front and rear motors or the fuel cell system and the battery, aiming to find their optimal distribution coefficients, respectively. Therefore, to fully exploit the energy-saving potential of the 4WD PFCEV, the electric–electric distribution of the front and rear motors, as well as the hydrogen–electric distribution of the fuel cell system and the battery, are necessary.

#### 3.1.1. M-ECMS for Front and Rear Motors

**Figure 6:**
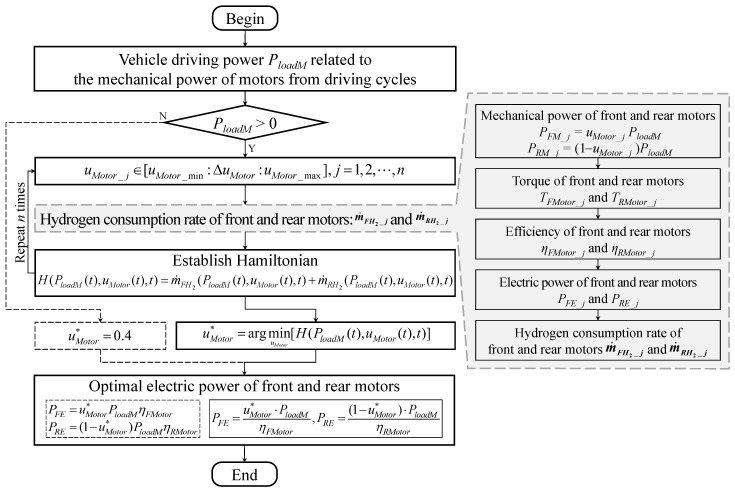
Power distribution between the front and rear motors.

In this paper, the flow of ECMS-based power distribution of the front and rear motors (M-ECMS) is shown in Figure 6. When the vehicle driving power is not greater than 0, i.e., when the vehicle is braking, the power distribution coefficient of the front and rear motors is a fixed value of 0.4. Conversely, when the vehicle is in a normal drive state, M-ECMS can minimize the total hydrogen consumption rate of the front and rear motors by ensuring their efficient operation. This study focuses on the normal drive state of the vehicle. Since the equivalent hydrogen consumption rates of the front and rear motors are related to their respective electric power, their relationship with the power distribution coefficient of the front and rear motors can be described as follows:

(18) $$\left\{\begin{array}{c} {\dot{m}}_{FH_{2}}\left(P_{loadM}\left(t\right),u_{Motor}\left(t\right),t\right)=\frac{\lambda_{Motor}P_{FE}\left(t\right)}{LHV_{H_{2}}}=\frac{\lambda_{Motor}P_{loadM}\left(t\right)u_{Motor}\left(t\right)}{LHV_{H_{2}}\eta_{FMotor}}\\ {\dot{m}}_{RH_{2}}\left(P_{loadM}\left(t\right),u_{Motor}\left(t\right),t\right)=\frac{\lambda_{Motor}P_{RE}\left(t\right)}{LHV_{H_{2}}}=\frac{\lambda_{Motor}P_{loadM}\left(t\right)\cdot \left(1-u_{Motor}\left(t\right)\right)}{LHV_{H_{2}}\eta_{RMotor}} \end{array}\right.$$

where ${\dot{m}}_{FH_{2}}$ and ${\dot{m}}_{RH_{2}}$ are the equivalent hydrogen consumption rates of the front and rear motors, respectively; $P_{FE}\left(t\right)$ and $P_{RE}\left(t\right)$ are the electric power of the front and rear motors at moment *t*, separately; $P_{loadM}\left(t\right)$ is the vehicle driving power at moment *t*; $u_{Motor}\left(t\right)$ is the power distribution coefficient between the front and rear motors at moment *t*; and $\lambda_{Motor}$ is the equivalent factor of hydrogen consumption of the motors. Since it is set to be the same for the front and rear motors, its initialized value does not affect the subsequent optimization solution.

Further, in order to achieve the minimum sum of hydrogen consumption rates of the front and rear motors, the optimized objective function of M-ECMS can be written as follows:

(19) $$J\left(t\right)=\int_{0}^{t_{0}}[{\dot{m}}_{FH_{2}}\left(P_{loadM}\left(t\right),u_{Motor}\left(t\right),t\right)+{\dot{m}}_{RH_{2}}\left(P_{loadM}\left(t\right),u_{Motor}\left(t\right),t\right)]dt$$

In order to solve for the minimum of the objective function expressed by the above equation, the Hamilton equation is established as follows:

(20) $$H\left(P_{loadM}\left(t\right),u_{Motor}\left(t\right),t\right)={\dot{m}}_{FH_{2}}\left(P_{loadM}\left(t\right),u_{Motor}\left(t\right),t\right)+{\dot{m}}_{RH_{2}}\left(P_{loadM}\left(t\right),u_{Motor}\left(t\right),t\right)$$

When the Hamilton equation takes the minimum value in a finite set, the corresponding control value is the optimal control input, and the expression is as follows:

(21) $$\begin{array}{c} u_{Motor}^{*}\left(t\right)=\arg\min_{u_{Motor}}\;\left[H\left(P_{loadM}\left(t\right),u_{Motor}\left(t\right),t\right)\right]\\ s.t.\left\{\begin{array}{c} u_{Motor\_\min}\left(t\right)\leq u_{Motor}\left(t\right)\leq u_{Motor\_\max}\left(t\right)\\ T_{FMotor\_\min}\left(t\right)\leq T_{FMotor}\left(t\right)\leq T_{FMotor\_\max}\left(t\right)\\ T_{RMotor\_\min}\left(t\right)\leq T_{RMotor}\left(t\right)\leq T_{RMotor\_\max}\left(t\right)\\ P_{FM\_\min}\left(t\right)\leq P_{FM}\left(t\right)\leq P_{FM\_\max}\left(t\right)\\ P_{RM\_\min}\left(t\right)\leq P_{RM}\left(t\right)\leq P_{RM\_\max}\left(t\right) \end{array}\right. \end{array}$$

where $u_{Motor}^{*}\left(t\right)$ is the optimal control input of power distribution coefficient between the front and rear motors at moment *t*; $u_{Motor\_\min}\left(t\right)$ and $u_{Motor\_\max}\left(t\right)$ are the minimum and maximum control input at moment *t*; $T_{FMotor}\left(t\right)$ and $T_{RMotor}\left(t\right)$ are the torques of the front and rear motors at moment *t*; $P_{FM}\left(t\right)$ and $P_{RM}\left(t\right)$ are the mechanical powers of the front and rear motors at moment *t*; $T_{FMotor\_\min}$ and $T_{FMotor\_\max}\left(t\right)$ are the minimum and maximum torques of the front motor at moment *t*; $T_{RMotor\_\min}$ and $T_{RMotor\_\max}\left(t\right)$ are the minimum and maximum torques of the rear motor at moment *t*; $P_{FM\_\min}\left(t\right)$ and $P_{FM\_\max}\left(t\right)$ are the minimum and maximum mechanical powers of the front motor at moment *t*; and $P_{RM\_\min}\left(t\right)$ and $P_{RM\_\max}\left(t\right)$ are the minimum and maximum mechanical powers of the rear motor at moment *t*.

Further, with the optimal control input of the power distribution coefficient, the electrical power of the front and rear motors can be written as follows:

(22) $$\left\{\begin{array}{c} P_{FE}\left(t\right)=\frac{u_{Motor}^{*}\left(t\right)\cdot P_{loadM}\left(t\right)}{\eta_{FMotor}}\\ P_{RE}\left(t\right)=\frac{\left(1-u_{Motor}^{*}\left(t\right)\right)\cdot P_{loadM}\left(t\right)}{\eta_{RMotor}} \end{array}\right.$$

where $P_{FE}\left(t\right)$ and $P_{RE}\left(t\right)$ are the electrical power of the front and rear motors at moment *t*, respectively. Thus, the vehicle demand power is calculated as follows:

(23) $$P_{load}\left(t\right)=P_{FE}\left(t\right)+P_{RE}\left(t\right)$$

where $P_{load}\left(t\right)$ is the vehicle demand power at moment *t*.

The power distribution coefficient between the front and rear motors solved by the M-ECMS can achieve optimal power distribution according to the different efficiency characteristics of the front and rear motors. Therefore, the proposed M-ECMS can achieve superior PFCEV economy.

#### 3.1.2. A-ECMS for Fuel Cells and Battery

**Figure 7:**
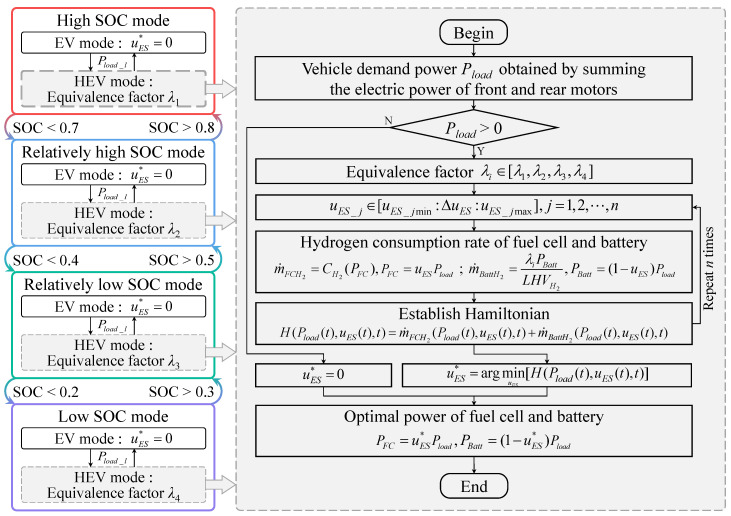
Power distribution between the fuel cell and the battery.

To further exploit the energy-saving potential of the 4WD PFCEVs, an adaptive ECMS (A-ECMS) is proposed in this study to solve the power distribution problem of the complex dual power sources, including the fuel cell system and the battery. A-ECMS follows the rule-based energy management framework, as shown in Figure 7. Similarly, A-ECMS is still divided into four levels according to battery SOC: high SOC, relatively high SOC, relatively low SOC, and low SOC, while each level is only classified into two levels according to the vehicle demand power: high power and low power. This reasoning emphasizes the fact that an excessive number of levels regarding the vehicle demand power could significantly increase the complexity of the method and is prone to the risk of over-constraining, which affects the solution of the optimal control process.

Similar to M-ECMS, the power distribution coefficient between the fuel cell system and the battery is set to 0, i.e., the demand power of the fuel cell system is 0 kW when the vehicle is braking or when the vehicle enters EV mode due to low vehicle demand power. This section similarly focuses on the drive state to achieve the optimal control input of power distribution with the minimum hydrogen consumption rate of the fuel cell system and the battery. The hydrogen consumption rate ${\dot{m}}_{FCH_{2}}$ of the fuel cell system and the equivalent hydrogen consumption rate ${\dot{m}}_{BattH_{2}}$ of the battery satisfy the following equation:

(24) $$\left\{\begin{array}{c} {\dot{m}}_{FCH_{2}}\left(P_{load}\left(t\right),u_{ES}\left(t\right),t\right)=C_{H_{2}}\left(P_{FC}\left(t\right)\right)=C_{H_{2}}\left(P_{load}\left(t\right)\cdot u_{ES}\left(t\right)\right)\\ {\dot{m}}_{BattH_{2}}\left(P_{load}\left(t\right),u_{ES}\left(t\right),t\right)=\frac{\lambda_{i}P_{Batt}\left(t\right)}{LHV_{H_{2}}}=\frac{\lambda_{i}P_{load}\left(t\right)\cdot \left(1-u_{ES}\left(t\right)\right)}{LHV_{H_{2}}} \end{array}\right.$$

where $P_{FC}\left(t\right)$ and $P_{Batt}\left(t\right)$ are the power of the fuel cell system and the battery at moment *t*, respectively; $u_{ES}\left(t\right)$ is the power distribution coefficient between the fuel cell system and the battery; $\lambda_{i}$ is the equivalent factor for converting the battery electrical energy consumption rate into hydrogen consumption rate and $\lambda_{i}\in \left[\lambda_{1},\lambda_{2},\lambda_{3},\lambda_{4}\right]$. Note that $\lambda_{1}$, $\lambda_{2}$, $\lambda_{3}$ and $\lambda_{4}$ correspond to the levels of high SOC, relatively high SOC, relatively low SOC, and low SOC, respectively. In the A-ECMS control framework, the dragonfly algorithm is employed to perform an iterative optimization search on the equivalent factor vector $\left[\lambda_{1},\lambda_{2},\lambda_{3},\lambda_{4}\right]$.

Similarly, the optimized objective function of A-ECMS can be expressed as the following equation:

(25) $$J\left(t\right)=\int_{0}^{t_{0}}[{\dot{m}}_{FCH_{2}}\left(P_{load}\left(t\right),u_{ES}\left(t\right),t\right)+{\dot{m}}_{BattH_{2}}\left(P_{load}\left(t\right),u_{ES}\left(t\right),t\right)]dt$$

To solve for the minimum of the objective function of the above equation, the Hamilton equation is established as follows:

(26) $$H\left(P_{load}\left(t\right),u_{ES}\left(t\right),t\right)={\dot{m}}_{FCH_{2}}\left(P_{load}\left(t\right),u_{ES}\left(t\right),t\right)+{\dot{m}}_{BattH_{2}}\left(P_{load}\left(t\right),u_{ES}\left(t\right),t\right)$$

Furthermore, in order to minimize the Hamilton equation in a finite set, the corresponding optimal control inputs can be written as follows:

(27) $$\begin{array}{c} u_{ES}^{*}\left(t\right)=\arg\min_{u_{ES}}\;\left[H\left(P_{load}\left(t\right),u_{ES}\left(t\right),t\right)\right]\\ s.t.\left\{\begin{array}{c} u_{ES\_\min}\left(t\right)\leq u_{ES}\left(t\right)\leq u_{ES\_\max}\left(t\right)\\ P_{FC\_\min}\left(t\right)\leq P_{FC}\left(t\right)\leq P_{FC\_\max}\left(t\right),P_{Batt\_\min}\left(t\right)\leq P_{Batt}\left(t\right)\leq P_{Batt\_\max}\left(t\right)\\ \lambda_{\min}\leq \lambda_{1}\leq \lambda_{\max},\lambda_{\min}\leq \lambda_{2}\leq \lambda_{\max},\lambda_{\min}\leq \lambda_{3}\leq \lambda_{\max},\lambda_{\min}\leq \lambda_{4}\leq \lambda_{\max} \end{array}\right. \end{array}$$

where $u_{ES}^{*}\left(t\right)$ is the optimal power distribution coefficient between the fuel cell system and the battery at moment *t*; $u_{ES\_\min}\left(t\right)$ and $u_{ES\_\max}\left(t\right)$ are the minimum and maximum of the power distribution coefficient at moment *t*; $P_{FC}\left(t\right)$, $P_{FC\_\min}\left(t\right)$, and $P_{FC\_\max}\left(t\right)$ are the demanded power of the fuel cell system and its minimum and maximum at moment *t*; $P_{Batt}\left(t\right)$, $P_{Batt\_\min}\left(t\right)$, and $P_{Batt\_\max}\left(t\right)$ are the demanded power of the battery and its minimum and maximum at moment *t*; and $\lambda_{\min}$ and $\lambda_{\max}$ are the minimum and maximum of equivalence factors, respectively.

Under the optimal control input, the demand power of the fuel cell system and the battery can be written as follows:

(28) $$\left\{\begin{array}{c} P_{FC}\left(t\right)=u_{ES}\left(t\right)\cdot P_{load}\left(t\right)\\ P_{Batt}\left(t\right)=\left(1-u_{ES}\left(t\right)\right)\cdot P_{load}\left(t\right) \end{array}\right.$$

Corresponding to the rule-based energy management framework, the maximum change in the output power of the fuel cell system is limited in this study, considering the impact of a wide range of fuel cell system power variations on its lifetime. Therefore, the amount of demand power change of the fuel cell system at moment *t* can be expressed as:

(29) $$\Delta P_{FC}\left(t\right)=u_{ES}\left(t\right)\cdot P_{load}\left(t\right)-P_{FC\_ACT}\left(t-1\right)$$

where $\Delta P_{FC}\left(t\right)$ is the amount of demand power change of the fuel cell system at moment *t*, and $P_{FC\_ACT}\left(t-1\right)$ is the actual output power of the fuel cell system at moment *t*− 1. Therefore, the actual output power of the fuel cell system and the battery at moment *t* is as follows:

(30) $$P_{FC\_ACT}\left(t\right)=\left\{\begin{array}{c} P_{FC\_ACT}\left(t-1\right)+\Delta P_{FC\_\max},\Delta P_{FC}\left(t\right)>\Delta P_{FC\_\max}\\ u_{ES}\left(t\right)\cdot P_{load}\left(t\right),\Delta P_{FC\_\min}\leq \Delta P_{FC}\left(t\right)\leq \Delta P_{FC\_\max}\\ P_{FC\_ACT}\left(t-1\right)+\Delta P_{FC\_\min},\Delta P_{FC}\left(t\right)<\Delta P_{FC\_\min} \end{array}\right.$$

(31) $$P_{Batt\_ACT}\left(t\right)=P_{load}\left(t\right)-P_{FC\_ACT}\left(t\right)$$

where $P_{FC\_ACT}\left(t\right)$ and $P_{Batt\_ACT}\left(t\right)$ are the actual output power of the fuel cell system and the battery at time *t*, respectively, and $\Delta P_{FC\_\min}$ and $\Delta P_{FC\_\max}$ are the minimum and maximum changes in the output power of the fuel cell system, respectively.

The proposed A-ECMS can theoretically improve the economy of the 4WD PFCEV by solving the optimal power distribution coefficient considering the fuel cell system and battery operating characteristics, which can further exploit the energy-saving potential of the 4WD PFCEV.

### 3.2. Dragonfly Algorithm for A-ECMS

In order to further improve the energy-saving performance of the PFCEV and achieve the purpose of operating condition adaptability, the dragonfly algorithm is employed to optimize the equivalent factor vector of A-ECMS, ensuring the minimal equivalent hydrogen consumption of the 4WD PFCEVs.

#### 3.2.1. Overview

The dragonfly algorithm [34] is a swarm intelligence optimization algorithm proposed in 2015. Similar to ant colony optimization (ACO) and particle swarm optimization (PSO), the dragonfly algorithm mathematically models the behavioral criteria of dragonfly populations, such as flight paths, avoidance of natural enemies, and food search, including separation, alignment, cohesion, target predation, and enemy avoidance. In order to establish the position update equation for each dragonfly individual, the neighborhood radius of the dragonfly individual should be defined, which can determine the update mode of each dragonfly individual’s position and enhance global optimality. Specifically, the radius of the neighborhood of each dragonfly individual can be expressed as:

(32) $$r_{i}\left(k\right)=\left(\frac{1}{4}+\frac{2k}{k\_\max}\right)\left(X_{\max}-X_{\min}\right)$$

where $r_{i}\left(k\right)$ is the neighborhood radius of the *i*-th dragonfly individual at the *k*-th iteration step; $X_{\min}$ and $X_{\max}$ are the minimum and maximum of the dragonfly individual’s position vector, respectively; and k_max is the maximum number of iterations. Note that the neighborhood radius increases with the number of iterations to improve the global convergence.

Based on the above five types of behavioral criteria, when there are other dragonfly individuals in the neighborhood of the *i*-th dragonfly individual, the position update equation for the *i*-th dragonfly individual is expressed as:

(33) $$X_{i}\left(k+1\right)=X_{i}\left(k\right)+\Delta X_{i}\left(k+1\right)$$

where $X_{i}\left(k+1\right)$ is the position vector of the (*k* + 1)-th generation of dragonfly individuals and $X_{i}\left(k\right)$ is the position vector of the *k*-th generation. $\Delta X_{i}\left(k+1\right)$ is the step vector of the (*k* + 1)-th generation, which is related to the above five types of behavioral criteria and satisfies the following equation:

(34) $$\Delta X_{i}\left(k+1\right)=\left(sS_{i}+aA_{i}+cC_{i}+tT_{i}+eE_{i}\right)+w\Delta X_{i}\left(k\right)$$

where $S_{i}$ and *s* represent the separation of the *i*-th individual and its weight, respectively; $A_{i}$ and *a* represent the alignment of the *i*-th individual and its weight, respectively; $C_{i}$ and *c* represent the cohesion of the *i*-th individual and its weight, respectively; $T_{i}$ and *t* represent the target predation of the *i*-th individual and its weight, respectively; $E_{i}$ and *e* represent the enemy avoidance of the *i*-th individual and its weight, respectively; and *w* is the inertia weight of the *i*-th individual. Note that *s*, *a*, and *c* are set as random numbers with respect to the number of iterations and that *t* is set as a random number to increase the global search ability. However, *e* and *w* are set to decrease with the number of iterations.

Further, separation avoids static collisions between each dragonfly and neighboring individuals. The action of separation can be calculated as follows:

(35) $$S_{i}=-\sum_{j=1}^{N}\left(X_{i}-X_{j}\right)$$

where $X_{i}$ is the current position of the *i*-th dragonfly individual, $X_{j}$ is the position of the *j*-th individual neighboring the *i*-th dragonfly individual, and *N* is the number of individuals neighboring the *i*-th dragonfly individual.

Alignment ensures that each dragonfly moves at the same speed as neighboring individuals. The action of alignment can be calculated as follows:

(36) $$A_{i}=\frac{1}{N}\sum_{j=1}^{N}V_{j}$$

where $V_{j}$ is the speed of the *j*-th individual neighboring the *i*-th dragonfly individual.

Cohesion ensures that each dragonfly tends to gather towards the mass center of the neighborhood. The action of cohesion can be calculated as follows:

(37) $$C_{i}=-X_{i}+\frac{1}{N}\sum_{j=1}^{N}X_{j}$$

Target predation is expressed as the attraction behavior of the dragonfly population to the food source. Specifically, the dragonfly individuals with the highest fitness can be considered as food, which can be calculated as follows:

(38) $$T_{i}=X_{Target}-X_{i}$$

where $X_{Target}$ is the position of target predation, i.e., the position of the dragonfly individual with the highest fitness.

Enemy avoidance is manifested as a distancing behavior of the dragonfly population from the enemy. Specifically, dragonfly individuals with the lowest fitness can be considered as the enemy, which can be calculated as follows:

(39) $$E_{i}=X_{Enemy}+X_{i}$$

where $X_{Enemy}$ is the position of enemy, i.e., the position of the dragonfly individual with the lowest fitness. To improve the randomness and global search ability of artificial dragonflies, when no other dragonfly individual exists neighboring the *i*-th dragonfly individual, the position of this individual is updated by the random Lévy flight, i.e., the position of the *i*-th dragonfly individual in the (*k* + 1)-th iteration is as follows:

(40) $$X_{i}\left(k+1\right)=X_{i}\left(k\right)+Lé\mathrm{vy}(\dim)\cdot X_{i}\left(k\right)$$

where dim is the dimension of the position vector of the dragonfly individual. In this study, the dimension is taken as 4, i.e., the Lévy flight of dragonfly individuals is limited to a 4D environment. Accordingly, the function of Lévy flight can be written as follows:

(41) $$Lé\mathrm{vy}(\dim)=\frac{0.01\times r_{1}}{{\left|r_{2}\right|}^{\frac{1}{\beta }}}\cdot {\left(\frac{\sin\left(\frac{\pi \beta }{2}\right)\cdot \beta !}{\beta \cdot 2^{\left(\frac{\beta -1}{2}\right)}\cdot \left(\frac{\beta -1}{2}\right)!}\right)}^{\frac{1}{\beta }}$$

where $r_{1}$ and $r_{2}$ are two random numbers in the range [0,1] and β is taken as a constant 1.5 in this study. Note that Lévy(dim) obtained from the above equation is a vector of dimension dim with the same elements.

#### 3.2.2. Equivalent Factor Optimization

Combined with the mathematical modeling process of the dragonfly algorithm above, the optimization process of the equivalent factor vector in A-ECMS for the power distribution problem between the fuel cell and the battery is defined as follows:

(1) Each dragonfly individual’s position vectors are considered a set of equivalent factor vectors, i.e., in the *k*-th iteration, the first element $X_{i}\left(k|1\right)$ in the position vector of the *i*-th dragonfly individual corresponds to the first element $\lambda_{1}$ of the equivalent factor vector in A-ECMS, and so on.
(2) In the optimization process, each element of the position vectors of all dragonfly individuals has a defined constraint that the dragonflies can only fly in a limited 4D environment, aiming to define the constraint of the equivalent factor vector in A-ECMS.
(3) The fitness of each dragonfly individual is characterized by the equivalent hydrogen consumption, which is equal to the sum of the hydrogen consumption of the fuel cell system and the battery. The smaller the equivalent hydrogen consumption, the higher the fitness of dragonfly individuals.

Therefore, optimization of A-ECMS equivalent factors based on the dragonfly algorithm (see Algorithm 1) is designed, and its specific implementation steps are as follows:

- Step 1: Generate a dragonfly population, i.e., initialize the relevant parameters in the dragonfly algorithm, including the number of dragonfly individuals, the maximum number of iterations, as well as the dimension, minimum, and maximum of the position vector of individuals.
- Step 2: Initialize the iteration count identifier to 1.
- Step 3: Initialize the position vectors of the first-generation dragonfly population, i.e., assign initial values to the equivalence factor vectors.
- Step 4: Calculate the fitness of each dragonfly individual and record the best individual fitness and its position vector in the current iteration, i.e., simulate based on the current equivalent factor vector and simple ECMS, feed back, and record the equivalent hydrogen consumption and equivalent factor vector for each simulation.
- Step 5: Update the position of the dragonfly population, i.e., specify the equivalent factor vector for the next iteration.
- Step 6: Add 1 to the iteration count identifier.
- Step 7: Determine the termination condition, i.e., determine whether the maximum number of iterations is reached. If the termination condition is met, move to Step 8, otherwise, return to Step 4.
- Step 8: Return the optimal individual fitness and its position vector within all iterations, i.e., the optimal equivalent hydrogen consumption and equivalent factor vector.

**Algorithm 1:** Optimization of A-ECMS equivalent factors based on dragonfly algorithm.**Input:** Number of dragonfly individuals *n*, maximum number of iterations k_max, individual position vector’s dimension dim, minimum $X_{\min}$, and maximum $X_{\max}$**Output:** Optimal position vector $X_{Target}$ and optimal fitness $F_{Target}$Initialize the iterative count identifier *k* to 1.Initialize position vectors of dragonfly populations $X_{i},i=1,2,\cdots ,n$.Initialize step vectors $\Delta X_{i},i=1,2,\cdots ,n$.**while** k≤k_max **do**      Calculate fitness of all dragonflies $F_{i},i=1,2,\cdots ,n$.      Update the position vector of target predation $X_{Target}$ with the highest fitness individual.      Update the position vector of enemy avoidance $X_{Enemy}$ with the lowest fitness individual.      Update the optimal fitness $F_{Target}$.      Update neighboring radius $r_{i},i=1,2,\cdots ,n$.      Update position vectors of all dragonflies $X_{i},i=1,2,\cdots ,n$.      k=k+1**end while****return** $X_{Target}$ and $F_{Target}$

In summary, the 4WD PFCEV can produce superior energy-saving performance through M-ECMS-based front and rear motor power distribution, and the A-ECMS-based fuel cell and battery power distribution combined to optimize the equivalent factors by the dragonfly algorithm.

## 4. Simulation and Discussion

To validate the comprehensive performance of the proposed A-ECMS, a series of simulation procedures are conducted based on MATLAB R2021b. The performance of energy management strategies including rule-based strategy (RB), equivalent consumption minimization strategy (ECMS), and adaptive equivalent consumption minimization strategy (A-ECMS) are compared under the driving cycle using five identical China light-duty vehicle test cycles (CLTC). Note that front and rear motor distributions in all EMSs are implemented based on M-ECMS. Methods for power distribution between the fuel cell system and the battery are categorized as RB, ECMS, and A-ECMS. All mentioned methods have a fixed simulation step of 0.01 s, and the initial battery SOC is set to 0.75. Simulations are performed on a computer with an Intel i5-6300HQ processor with 8 GB memory.

The illustrations for RB, ECMS, and A-ECMS are as follows:

- RB: A rule-based EMS can competently distribute the power of the fuel cell and the battery. Twelve fuel cell system demand power levels are determined by dividing the battery SOC into four levels: high SOC, relatively high SOC, relatively low SOC, and low SOC, as well as dividing the vehicle demand power into three levels: high, medium, and low. Note that the maximum power $P_{FC\_\max}$, the efficient operating point power $P_{FC\_opt}$, and the idle power $P_{FC\_idle}$ of the fuel cell system are set to 50, 20, and 2 kW, respectively.
- ECMS: A power distribution strategy for the fuel cell and the battery based on equivalent consumption minimization. Specifically, the instantaneous optimal demand power of the fuel cell system is determined by minimizing the equivalent hydrogen consumption based on the framework of four SOC levels divided by RB and the vector, including four equivalent factors. Note that the EV and HEV mode switching thresholds are set to [30 kW, 40 kW] at the high SOC and relatively high SOC levels and [0 kW, 15 kW] at the lower SOC and relatively low SOC levels. The equivalent factor vector $\left[\lambda_{1},\lambda_{2},\lambda_{3},\lambda_{4}\right]$ in this method is set to fixed values of [2.73, 2.73, 2.73, 2.73].
- A-ECMS: A power distribution strategy for the fuel cell and the battery based on adaptive equivalent fuel consumption minimization. In contrast to ECMS, A-ECMS has a variable equivalent factor vector, and the dragonfly algorithm is used to find an optimized combination of equivalent factors with minimal equivalent hydrogen consumption.

### 4.1. General Comparison of Different EMSs

A-ECMS with the dragonfly algorithm’s optimized equivalent factor vector can theoretically deliver a more competitive energy-saving performance. To validate this argument, the simulation results shown in Table 2 are obtained under the baseline of RB and ECMS and the economic evaluation criterion of the equivalent hydrogen consumption during normal vehicle driving. All three EMSs have the same initial SOC of 0.75. However, RB has the lowest ending SOC (0.357), while A-ECMS has the highest one (0.399). The equivalent hydrogen consumption is defended as the summation of the hydrogen consumption of the fuel cell system and the equivalent hydrogen consumption of the battery, whose results are shown as follows: the method with the lowest equivalent hydrogen consumption is A-ECMS with 757.768 g, followed closely by ECMS with 768.462 g, and RB with the highest equivalent hydrogen consumption of 773.346 g. Macroscopically, A-ECMS holds the highest energy-saving optimality, accounting for 2.01%.

**Figure 8:**
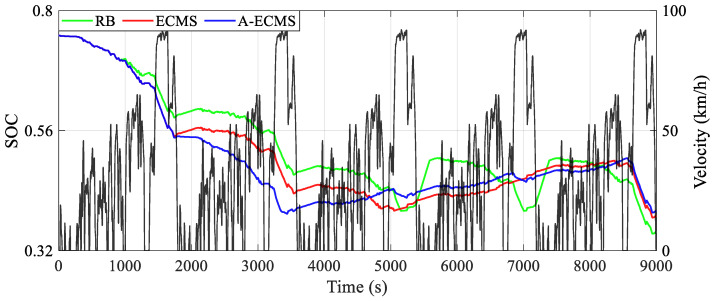
Curves of battery SOC using different methods.

The curves of battery SOC using different methods during the 9000 s simulation are shown in Figure 8. All three methods have almost the same curve of SOC decrease until about 1000 s, since all of them tend to respond to the vehicle demand power individually by using the battery at the high SOC level. In the subsequent range from approximately 1000 s to 1800 s, RB exhibits a slower rate of decline than the other two methods. This is because the fuel cell system in RB starts and begins to provide power after reaching the threshold of 0.7 for switching from the high SOC level to the relatively high SOC level. Before reaching the threshold of 0.4 for switching to the relatively low SOC level, i.e., until about 3500 s, all three methods show a gradual decrease in SOC over the low-speed range and a rapid decrease over the high-speed range. After that, A-ECMS reaches the threshold of 0.4 at about 3500 s and then switches to the relatively low SOC level until 8500 s. During this process, the battery SOC increases slowly and linearly, which indicates that the fuel cell system is limited in charging the battery. However, ECMS enters the relatively low SOC level only at about 5000 s, but the linear SOC growth is significantly faster than A-ECMS, especially between 6500 s and 7500 s. As for RB, after switching to the relatively low SOC level (around 5200 s), the battery SOC rises rapidly and frequently switches between the relatively low and relatively high SOC levels. It indicates that the fuel cell system provides higher or even excess power for the battery, resulting in its over-charging and over-discharging possibility. Finally, after reaching about 8500 s, the curves of the three methods fall rapidly and almost simultaneously under the influence of the high vehicle demand power due to high vehicle velocity.

**Figure 9:**
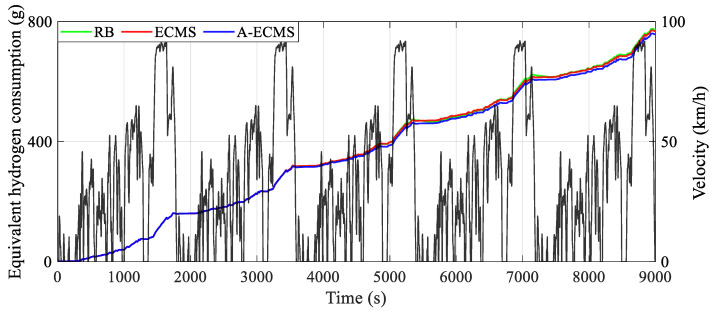
Curves of equivalent hydrogen consumption using different methods.

Corresponding to the battery SOC curves, Figure 9 demonstrates the curves of equivalent hydrogen consumption obtained by different methods. The diagram shows that the equivalent hydrogen consumption curves are essentially the same for about the first 1800 s and even in the time range of about the first 3500 s. However, A-ECMS is the first to switch to the relatively low SOC level after 3500 s, leading to a significantly slower increase in both hydrogen consumption and battery SOC than the other two methods under a relatively balanced distribution relationship between the power of the fuel cell system and the battery. Furthermore, as for RB, the relatively unbalanced power distribution relationship of the power of the fuel cell system and the battery leads to both the dramatic drop of battery SOC between 5000 s and 5200 s and the dramatic rise of battery SOC after about 5000 s and about 7000 s, which results in a noticeable increase in energy consumption. In addition, as RB re-switches to the relatively high SOC level after 5500 s, the power distribution back to a balanced relationship decreases the SOC, which in turn leads to a slower increase in hydrogen consumption. Therefore, the unbalanced power distribution between the fuel cell system and the battery is the main factor in the different hydrogen consumption, i.e., a balanced relationship of power distribution facilitates the release of energy-saving potential.

### 4.2. Comparison of Component Operating States with Different EMSs

**Figure 10:**
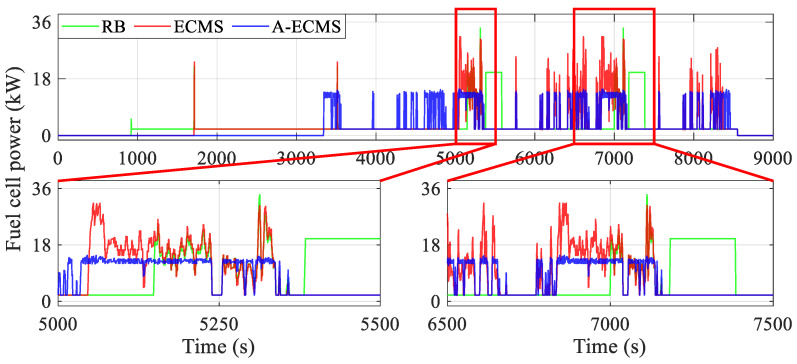
Curves of fuel cell system power using different methods.

**Figure 11:**
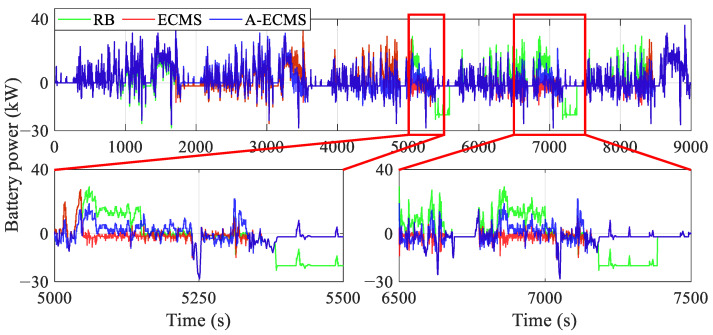
Curves of the battery power using different methods.

**Figure 12:**
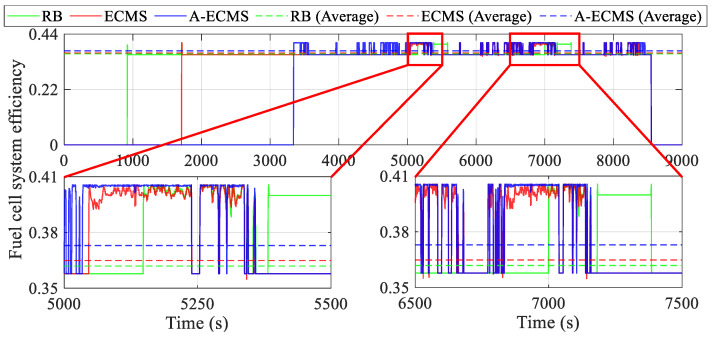
Curves of the fuel cell system efficiency using different methods.

**Figure 13:**
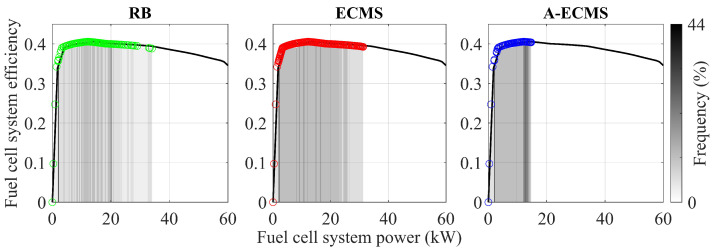
Operating points of the fuel cell system using different methods.

**Figure 14:**
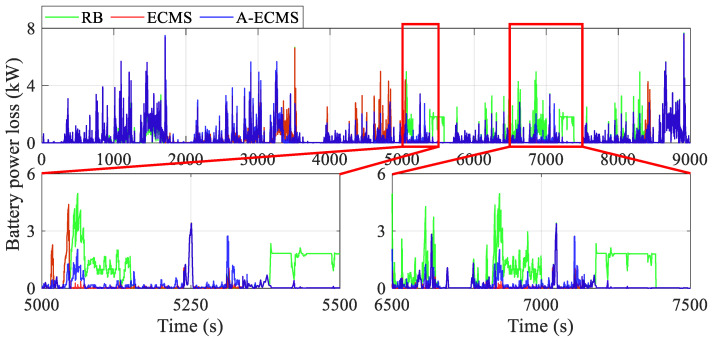
Curves of the battery power loss using different methods.

**Figure 15:**
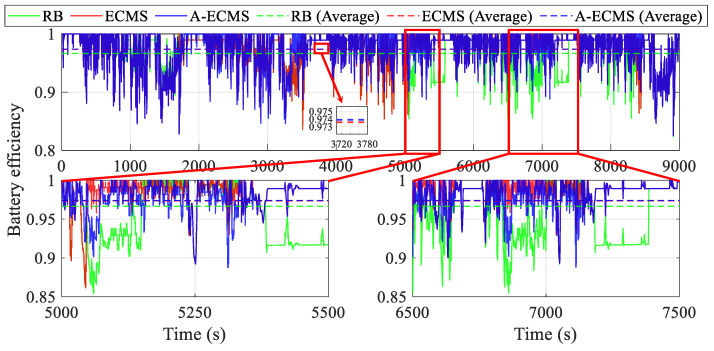
Curves of the battery efficiency using different methods.

To further dissect the mechanism of the power distribution of the fuel cell system and the battery on the equivalent hydrogen consumption, this section analyzes the operating states of the components, especially the power and efficiency of the fuel cell system and the battery, etc., as shown in Figure 10, Figure 11, Figure 12, Figure 13, Figure 14 and Figure 15.

Figure 10 and Figure 11 present the curves of the fuel cell system power and the battery power under different methods, respectively. The RB’s fuel cell system starts as early as less than 1000 s and then is used to fixed point work or respond to vehicle demand power, while A-ECMS starts up the latest (around 3300 s). Furthermore, the curves show that the fuel cell system power of RB and ECMS is generally higher than that of A-ECMS, even reaching twice as much as it does between 5000 s and 8500 s. This directly leads to a lower battery charging power of A-ECMS during this period, which reduces the power loss due to overcharging of the battery. Additionally, A-ECMS ensures that the fuel cell system operates at a uniform point of power, mainly concentrated near the high-efficiency region (about 14 kW), which guarantees the efficient operation of the fuel cell system. In addition, the magnified diagram from 5000 s to 5500 s and from 6500 s to 7500 s in Figure 11, especially the curves around 5100 s, 5400 s, 6900 s, and 7300 s, also shows that the fixed-point operating fuel cell system in RB tends to have two extremes, i.e., over-discharging and over-charging of the battery, which leads to considerable power loss caused by internal resistance. ECMS, on the other hand, reflects another extreme, i.e., the fuel cell system mainly responds to the vehicle demand power. Although a lower battery power is beneficial for reducing power loss, it will seriously affect the possibility of operating in the high-efficiency region and even bring unnecessary degradation due to frequent power fluctuations of the fuel cell system. The above discussion echoes the conclusions in the section on the general comparison.

Figure 12 illustrates the variation of the fuel cell system efficiency with time to further analyze the reason behind the better energy consumption of A-ECMS. After starting the fuel cell system, the system efficiency of all three methods is maintained in the range of 0.35 to 0.41. It can be seen from the two magnified diagrams spanning 500 s and 1000 s, respectively, that once the fuel cell system of the A-ECMS is taken off idle, it remains near the high efficiency point. However, both ECMS, which tries to respond to most of the vehicle demand power, and RB, which over-charges and over-discharges the battery, fail to keep the fuel cell system operating at high efficiency. Excluding the fuel cell system shutdown, A-ECMS occupied the highest average fuel cell system efficiency at 0.3729, followed closely by ECMS at 0.3649. The lowest average fuel cell system efficiency is RB at 0.3619.

In order to visualize the influence of the operating points of the fuel cell system on the energy-saving potential, Figure 13 illustrates the frequency distribution of the operating points by different methods. A-ECMS ensures that most operating points are near the highest efficiency point, bringing lower energy consumption. In addition, ECMS has the widest distribution of the fuel cell system operating points, thus taking up most of the work of responding to the vehicle demand power. In contrast, RB works more on fixed operating points obtained by hierarchical division. Therefore, the energy-saving potential of both ECMS and RB has to be explored.

Figure 14 and Figure 15 demonstrate the curves of battery power loss and battery efficiency, respectively. The magnified diagrams from 5000 s to 5500 s and from 6500 s to 7500 s demonstrate that the battery power loss due to internal resistance is generally lower due to the lower battery charging and discharging power of the ECMS, but this energy consumption advantage cannot compensate for the negative impact due to the unfavorable operating condition of the fuel cell system. More dramatically, the over-charging and over-discharging of the battery make the RB’s fuel cell system work at a suboptimal point and introduce more internal resistance and loss of efficiency. Assuming a battery efficiency of 1 at the power of 0 kW, A-ECMS has the highest average battery efficiency (0.9741), followed by ECMS (0.9736), and RB has the lowest (0.9648). Although the battery efficiency of A-ECMS is not significantly better than that of ECMS, the efficient operation of the fuel cell system of A-ECMS brings a more competitive overall energy-saving performance.

Therefore, the unbalanced power distribution brings about unreasonable operating points of the fuel cell, and over-charging or over-discharging of the battery will directly affect their working efficiency and unnecessary power loss, leading to higher equivalent hydrogen consumption. Conversely, a balanced power distribution allows the power components to work in the efficient zone and reduces energy losses, improving vehicle economy.

## 5. Conclusions

This paper proposes a novel A-ECMS energy management strategy for a 4WD PFCEV to improve fuel economy and operating condition adaptability. The power distribution between the front and rear motors is realized by ECMS, considering the optimal control under different motor characteristics. In addition, an A-ECMS with variable equivalent factors based on the dragonfly algorithm is proposed under a hierarchical energy management framework to exploit the energy-saving potential and optimize the adaptation to operating conditions through optimal control of the fuel cell system and battery. Moreover, the superior performance of A-ECMS is validated at the baseline for RB and ECMS. The energy-saving optimality of the optimal A-ECMS is improved by 2.01% compared to the above baseline.

## Figures and Tables

**Table 1:** Basic parameters of the studied PFCEV.

Characteristic	Value
Mass	1860 kg
Tire rolling radius	350 mm
Rolling resistance coefficient	0.015
Air drag coefficient	0.3
Frontal area	2 m${}^{2}$
Front motor speed and torque range	0–14,000 rpm/−137–137 Nm
Rear motor speed and torque range	0–10,000 rpm/−195–195 Nm
Battery nominal voltage	362 V
Battery capacity	40 Ah/14.48 kWh
Maximum net power of fuel cell	60 kW

**Table 2:** General comparison of the simulation results under different EMSs.

EMSs	Initial SOC	Ending SOC	HC of the Fuel Cell System ^1^	EHC of the Battery ^2^	EHC	Energy-Saving Optimality
RB	0.75	0.357	479.190 g	294.156 g	773.346 g	0 %
ECMS	0.75	0.389	487.518 g	280.945 g	768.462 g	0.63 %
A-ECMS	0.75	0.399	484.731 g	273.037 g	757.768 g	2.01 %

^1^ HC represents hydrogen consumption. ^2^ EHC represents equivalent hydrogen consumption.

## Data Availability

Not applicable.
